# Nomogram for Osteoporosis Risk Using LDCT Trabecular Parameters

**DOI:** 10.3390/diagnostics16101429

**Published:** 2026-05-08

**Authors:** Pin-Chieh Wu, Yun-Ju Wu, Chiao-Lin Hsu, Hsien-Chung Yu, Chi-Shen Chen, Fu-Zong Wu

**Affiliations:** 1 Health Management Center, Kaohsiung Veterans General Hospital, Kaohsiung 813414, Taiwan; 2 Department of Pharmacy, Chia Nan University of Pharmacy and Science, Tainan 717301, Taiwan; 3 Department of Radiology, Kaohsiung Veterans General Hospital, Kaohsiung 813414, Taiwan; 4 Center for Geriatrics and Gerontology, Kaohsiung Veterans General Hospital, Kaohsiung 813414, Taiwan; 5 Faculty of Medicine, School of Medicine, National Yang Ming Chiao Tung University, Taipei 112304, Taiwan; 6 Faculty of Clinical Medicine, National Yang Ming Chiao Tung University, Taipei 112304, Taiwan

**Keywords:** osteoporosis, nomogram, LDCT, trabecular bone

## Abstract

**Background:** Low-dose computed tomography (LDCT) offers a unique opportunity to assess osteoporosis risk during routine lung cancer screening. This study aims to develop an integrated prediction model using trabecular bone features from LDCT and clinical factors to identify high-risk individuals early. **Methods:** This retrospective observational cohort study included 429 adults who underwent both DEXA and LDCT scans within one week at Kaohsiung Veterans General Hospital (2018–2022). Clinical data, including demographics, lifestyle factors, and comorbidities, were extracted from electronic records. Participants were categorized into osteoporotic (T-score ≤ −2.5) and non-osteoporotic groups. Trabecular bone morphometry was assessed at the T12 vertebra using QUIBIM Precision^®^ software, analyzing parameters such as BV/TV, Tb.Th, Tb.N, Tb.Sp, D2D, D3D, and QTS. ROI placement and measurements followed standardized protocols. Ethical approval was obtained, and informed consent was waived. Statistical analyses included t-tests, ROC curves, logistic regression, and Delong tests to compare clinical and trabecular predictors of osteoporosis using SPSS v22. **Results:** In this study of 429 individuals, osteoporosis was significantly associated with female gender, older age, lower BMI, and smaller waist circumference. Trabecular bone morphometry revealed that osteoporotic individuals had significantly thinner trabeculae (lower Tb.Th), higher trabecular number (Tb.N), and more complex trabecular architecture (higher D2D/D3D), with lower QTS. Logistic regression showed that Model 1—the model combining clinical factors and Tb.N—showed a slightly higher predictive performance (AUC 0.738) than Model 2 (AUC 0.711), although the improvement was modest (*p* = 0.022). A nomogram based on age, sex, BMI, waist circumference, and Tb.N effectively estimated osteoporosis probability, providing a clinically useful tool for risk stratification. **Conclusions:** In conclusion, combining trabecular bone morphometry (Tb.N) from routine LDCT with age, sex, BMI, and waist circumference enhances osteoporosis risk prediction, enabling personalized assessment without extra radiation during standard lung cancer screening.

## 1. Introduction

Low-dose computed tomography (LDCT) is vital for lung cancer screening, enabling early detection and intervention [1]. In the context of holistic care, LDCT screening’s widespread use also offers opportunities to assess other health conditions, including osteoporosis and cardiovascular risk, through detailed bone structure and coronary calcium score analysis [2]. Although dual-energy X-ray absorptiometry (DEXA) is a reliable tool for diagnosing osteoporosis, its accessibility remains limited [3]. Therefore, identifying high-risk populations for osteoporosis and providing them with subsequent diagnostic confirmation and care has become an important public health issue in aging societies.

In a recent study, we identified age, height, weight, and BMI as key variables for our osteoporosis prediction model and assessed its performance using the area under the receiver operating characteristic curve (AUC). The artificial neural network model significantly outperformed the Osteoporosis Self-Assessment Tool for Asians model and other machine learning algorithms in both men and women (AUC: 0.67 for men; 0.77 for women), and demonstrated consistent performance across different populations [4]. However, bone mechanical competence is also governed by trabecular micro-architecture; its integrity depends on several interrelated attributes, including trabecular thickness, number, connectivity, and overall volume [5]. Research that integrates such micro-architectural metrics with clinical variables in osteoporosis risk models remains sparse, and the predictive performance of these combined models has yet to be thoroughly evaluated.

The rationale for the present study is to address this gap by developing an integrated prediction model that incorporates trabecular bone morphometric features extracted from LDCT scans alongside clinical factors. By using data from LDCT lung cancer screening, this integrated approach aims to facilitate the early identification and management of individuals at high risk for osteoporosis within a population already undergoing routine imaging.

## 2. Materials and Methods

### 2.1. Study Design and Participants

This retrospective observational cohort study examined individuals aged 20 years or older who underwent DEXA at the Kaohsiung Veterans General Hospital in Taiwan. Participants were included if both a DEXA scan and an LDCT scan were performed within one week. All chest LDCT scans were performed using a 256-slice CT scanner (Revolution CT, GE Healthcare, Milwaukee, WI, USA), covering the region from the lung apex to the lung base. Scans were obtained during full inspiration without the administration of contrast agents. The scanning protocols across different vendors were generally comparable and included a tube voltage of 120 kVp, a slice thickness of 1–2.5 mm, and reconstruction using a soft-tissue kernel.

**Figure 1:**
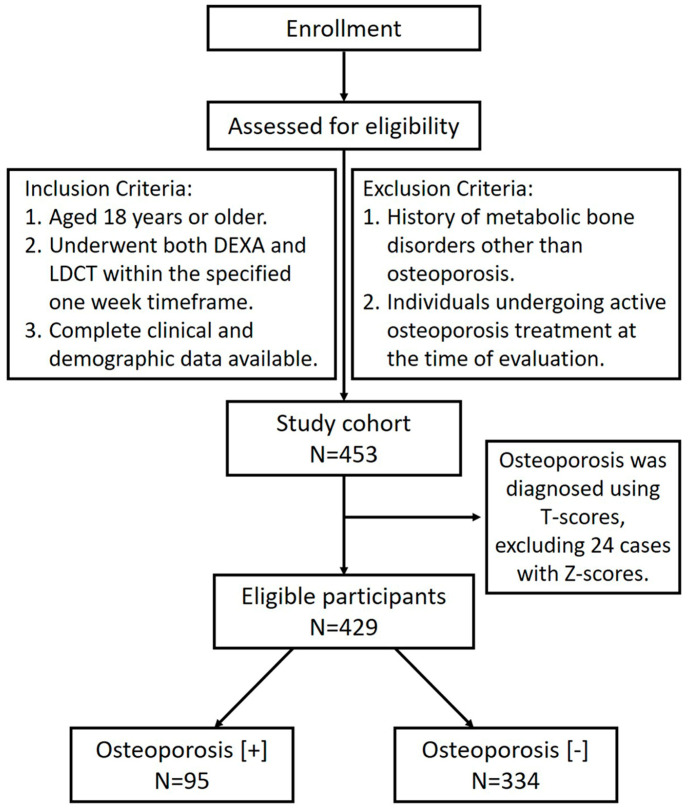
Flowchart of LDCT Participants Based on Enrollment Criteria and Osteoporosis Status.

Inclusion criteria were as follows: participants aged ≥18 years who underwent both DEXA and LDCT within a one-week interval, with complete clinical and demographic data available. Exclusion criteria included patients with a history of metabolic bone disorders other than osteoporosis and individuals undergoing active osteoporosis treatment at the time of evaluation. Between January 2016 and December 2022, a total of 510 individuals were enrolled. Of these, 57 were excluded due to incomplete or missing key data, and 24 were excluded for having Z-scores instead of T-scores, leaving a final cohort of 429 participants for analysis, shown in Figure 1. The study drew upon clinical and demographic data obtained from electronic medical records. The collected information included demographic characteristics such as age, gender, body mass index (BMI), body fat percentage, and waist circumference. Renal function markers, including blood urea nitrogen (BUN), serum creatinine, and estimated glomerular filtration rate (eGFR), were also recorded. Additionally, lifestyle factors such as smoking status (categorized as never/passive, former smoker, or current smoker), alcohol consumption, and physical activity levels were assessed. Relevant comorbidities, including cardiovascular disease, thyroid disease, cancer history, autoimmune disease, and diabetes mellitus, were also documented. A total of 429 eligible participants were categorized into two groups based on osteoporosis status: non-osteoporotic (Osteoporosis [−], N = 334) and osteoporotic (Osteoporosis [+], N = 95). Osteoporosis was diagnosed based on T-scores from DEXA scans, with a T-score of ≤−2.5 used to define osteoporosis according to World Health Organization (WHO) criteria [6]. This study was reviewed and approved by the Institutional Review Board of Kaohsiung Veterans General Hospital (approval number: KSVGH22-CT11-17). As it involved retrospective analysis of anonymized data, the requirement for informed consent was waived. The study was carried out in accordance with the ethical principles outlined in the Declaration of Helsinki.

### 2.2. Measurement of Trabecular Bone Morphometry

For trabecular bone analysis, the T12 vertebral body was selected as the region of interest (ROI). A circular ROI with an area of 1.5 cm^2^ was placed in the axial view of the vertebral body (central portion). T12 was selected because it is consistently included in routine low-dose chest CT (LDCT) examinations, providing a practical and reproducible level for opportunistic assessment. Its trabecular structure can be clearly visualized, enabling reliable ROI placement and quantitative analysis, thereby supporting its use as a standardized vertebral level for trabecular evaluation. Interobserver reproducibility was assessed in 30 randomly selected cases, yielding an intraclass correlation coefficient (ICC) of 0.910, indicating excellent agreement. Consistent with our findings, previous studies have also reported high reproducibility for vertebral trabecular measurements, with ICC values ranging from 0.933 to 0.994 (all *p* < 0.001) [7].

**Figure 2:**
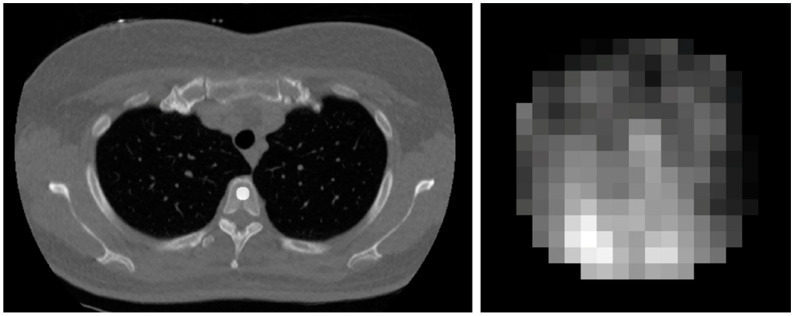
Trabecular bone analysis was performed on the T12 vertebral body, which was selected as the region of interest (ROI). A circular ROI of 1.5 cm^2^ was placed centrally in the axial view. The volume of interest (VOI) encompassed five consecutive slices, each 2 mm thick, totaling 1 cm in thickness.

The analysis covered a defined volume of interest (VOI) with a total slice thickness of 1 cm, consisting of five slices, each 2 mm thick, shown in Figure 2. Trabecular bone microstructure was quantitatively analyzed using QUIBIM Precision^®^ software (version 2.8, QUIBIM S.L., Valencia, Spain). The following parameters were extracted: bone volume fraction (BV/TV, %), trabecular thickness (Tb.Th, mean, µm), trabecular number (Tb.N, arbitrary units [au]), trabecular separation (Tb.Sp, mean, µm), two-dimensional and three-dimensional fractal dimensions (D2D and D3D), and the quantitative trabecular score (QTS).

BV/TV represents the proportion of bone volume relative to the total volume of the region of interest. In QUIBIM, the reconstructed trabecular bone is composed of numerous small voxels; accordingly, BV/TV is calculated as the ratio of voxels identified as bone (n_voxel of BV) to the total number of voxels within the volume of interest (n_voxel of TV). Tb.Th is evaluated by analyzing axial slices, in which each trabecular structure is depicted by multiple contours forming a tree-like branching pattern composed of numerous skeletal lines. Tb.Th is calculated by first measuring the minimum two-dimensional width (2D width) of each skeletal line, followed by averaging these widths across all skeletal lines and contours. Tb.N is derived as the ratio of BV/TV to Tb.Th, estimating the number of trabeculae per unit length. Tb.Sp reflects the average distance between trabeculae, while its standard deviation indicates the variability in spacing. Additionally, the D2D and D3D provide measures of the complexity and structural irregularity of the trabecular network in 2D and 3D space, respectively. The QTS, a built-in parameter in the QUIBIM trabecular analysis module, serves as an overall index of trabecular bone quality. All measurements were performed in a standardized manner to ensure consistency in ROI placement and parameter extraction.

### 2.3. Statistical Analysis

The statistical analysis was performed using SPSS Statistics Version 22.0 (IBM Corp., Armonk, NY, USA). Descriptive statistics were calculated for all variables, and continuous variables were presented as means ± standard deviations (SD), while categorical variables were expressed as frequencies and percentages.

An independent t-test was used to compare clinical and trabecular bone morphometry parameters between patients with and without osteoporosis. To assess multicollinearity among the parameters, a Variance Inflation Factor (VIF) test was conducted. Receiver operating characteristic (ROC) curve analysis was conducted to evaluate the discriminatory ability of individual trabecular bone parameters for osteoporosis. Two multivariable logistic regression models were built. Model 1 combined the screened clinical covariates with the trabecular bone morphometric parameter that exhibited the strongest univariable discriminatory power, whereas Model 2 contained only the screened clinical covariates. Model performance will be quantified by the AUC. Statistical significance is defined as *p* < 0.05. Finally, ROC curve analysis was performed, and the AUC was calculated to compare the predictive performance of the two logistic regression models. The Delong test assessed AUC differences to determine significant predictive differences between models for osteoporosis status. With a study cohort of 429 individuals, post hoc analysis indicated sufficient statistical power. Missing data accounted for ≤4% of all observations and appeared to occur at random.

## 3. Results

### 3.1. Study Population Characteristic

Table 1 presents the clinical characteristics of a study cohort of 429 individuals, categorized based on osteoporosis status: osteoporosis-negative (N = 334) and osteoporosis-positive (N = 95). Among the cohort, 31.2% are female and 68.8% are male, with osteoporosis significantly more common in females (32.1% vs. 17.6%, *p* = 0.001). The osteoporosis group has a higher mean age (68.13 ± 8.030 vs. 63.83 ± 8.101 years, *p* < 0.001). BMI (23.51 ± 3.1 vs. 25.67 ± 3.48 kg/m^2^, *p* < 0.001) and waist circumference (85.19 ± 10.359 vs. 89.99 ± 9.174 cm, *p* < 0.001) are lower in individuals with osteoporosis, while body fat percentage does not differ significantly (*p* = 0.287). Biochemical markers, including BUN (*p* = 0.543), creatinine (*p* = 0.683), and eGFR (*p* = 0.373), show no significant differences. Lifestyle factors such as smoking (*p* = 0.574), alcohol consumption (*p* = 0.428), and exercise habits (*p* = 0.748) also do not significantly differ between groups. Regarding medical history, there are no significant differences in the prevalence of cardiovascular disease (*p* = 0.197), thyroid disease (*p* = 0.311), cancer history (*p* = 0.572), autoimmune disease (*p* = 0.291), or diabetes mellitus (*p* = 0.086). Overall, osteoporosis is associated with female gender, older age, lower BMI, and lower waist circumference, while other lifestyle and medical factors appear unrelated.

### 3.2. Trabecular Bone Morphometry in the Study Population

Table 2 shows that osteoporotic individuals have significantly reduced trabecular thickness (Tb.Th, *p* = 0.004) but an increased trabecular number (Tb.N, *p* = 0.001), indicating an increased count of thinner struts. Both 2D (D2D, *p* = 0.015) and 3D fractal dimensions (D3D, *p* = 0.003) are significantly elevated, suggesting greater structural complexity. Additionally, the QTS is notably lower in the osteoporosis group (*p* = 0.002), reflecting a decline in overall trabecular integrity. However, bone volume fraction (BV/TV, *p* = 0.418) and trabecular separation (Tb.Sp, *p* = 0.222) do not show significant differences, indicating comparable bone volume and trabecular spacing between groups.

### 3.3. Logistic Regression Model for Osteoporosis Prediction

The logistic regression analysis in Table 3 evaluates two models for predicting osteoporosis: Model 1, which integrates both clinical parameters and Tb.N—the variable with the highest univariable discriminatory power, as indicated by its AUC of 0.612 (Table 4)—was compared with Model 2, which relies solely on clinical variables. In Model 1, age is a significant predictor (OR = 1.07, 95% CI: 1.037–1.104, *p* < 0.001), indicating an increasing likelihood of osteoporosis with age. Gender (OR = 0.571, 95% CI: 0.325–1.002, *p* = 0.051) shows a trend toward significance, suggesting males may have a lower risk, though not statistically significant. BMI (OR = 0.829, 95% CI: 0.722–0.951, *p* = 0.008) is negatively associated with osteoporosis, implying a protective effect, while waist circumference (OR = 1.019, 95% CI: 0.967–1.075, *p* = 0.478) is not significantly associated. Tb.N, a trabecular bone morphometry parameter with the highest value, was highly significant (OR = 19,062.087, 95% CI: 22.233–16,343,266.88, *p* = 0.004), indicating a detrimental micro-architectural shift attributable to significant trabecular thinning despite no significant change in BV/TV. The overall fit of Model 1 is strong, with an overall odds ratio (OR = 299.455, 95% CI: 63.133–1420.388, *p* < 0.001), suggesting that integrating trabecular bone morphometry significantly enhances predictive performance. In Model 2, age remains a significant predictor (OR = 1.06, 95% CI: 1.029–1.092, *p* < 0.001), reinforcing its association with osteoporosis risk. Gender (OR = 0.517, 95% CI: 0.298–0.898, *p* = 0.019) is statistically significant, indicating females have a higher osteoporosis risk. BMI (OR = 0.832, 95% CI: 0.727–0.952, *p* = 0.007) remains protective, while waist circumference (OR = 1.01, 95% CI: 0.959–1.063, *p* = 0.714) does not show a significant effect. The overall performance of Model 2 (OR = 366.701, 95% CI: 70.006–1920.821, *p* < 0.001) suggests strong predictive power but slightly lower robustness compared to Model 1.

### 3.4. Assessment of the Performance of the Different Models for Osteoporosis

Table 4 evaluates the predictive performance of different models and trabecular bone morphometry variables for osteoporosis, including statistical metrics such as the AUC, 95% confidence intervals (CI), and *p*-values. The results show that Tb.Th has the lowest AUC (0.362), indicating poor predictive performance, while Tb.N has an AUC of 0.612 with statistical significance (*p* = 0.001). The 2D and 3D fractal dimensions (D2D, D3D) have AUCs of 0.585 and 0.609, respectively, indicating moderate predictive ability, whereas the QTS has an AUC of 0.367, showing weak predictive performance. Regarding predictive models, Model 1 has the highest AUC (0.738), demonstrating the best predictive capability, while Model 2 has a slightly lower AUC (0.711) but remains statistically significant. Comparing Model 1 and Model 2, the AUC difference is 0.0273, with a standard error (SE) of 0.0119, a confidence interval of 0.00393–0.0506, and a *p*-value of 0.022, indicating that Model 1 is significantly superior to Model 2. Overall, among trabecular bone morphometry variables, Tb.N shows better predictive performance, while predictive models outperform individual trabecular parameters, with Model 1 demonstrating the highest predictive ability. Sensitivity analysis using randomly sampled balanced (95/95) and imbalanced (95/334) datasets showed consistent performance, indicating model robustness to class imbalance. The balanced model achieved an AUC of 0.751 (95% CI: 0.683–0.820, *p* < 0.001), demonstrating significant discriminative ability for osteoporosis diagnosis.

### 3.5. Logistic-Based Nomogram for Osteoporosis Prediction

**Figure 3:**
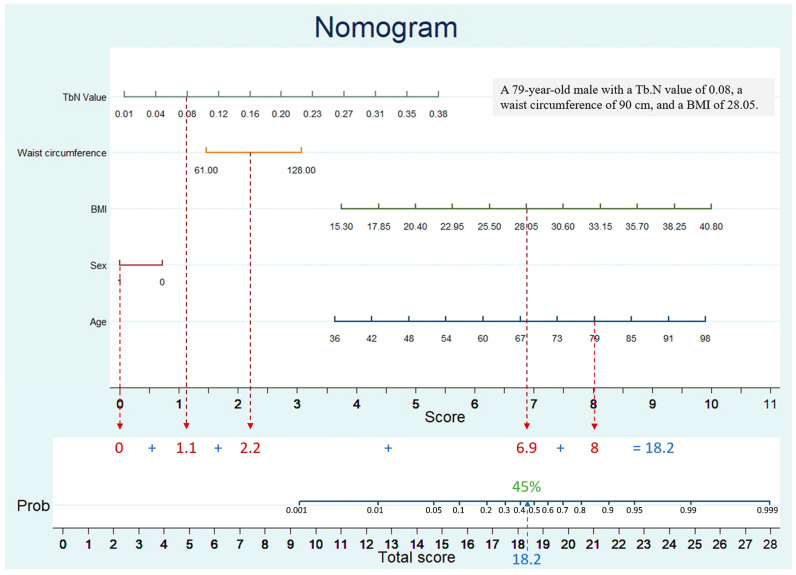
Nomogram for Predicting Osteoporosis Risk Based on TbN, Waist Circumference, BMI, Sex, and Age.

The probability of osteoporosis in the study cohort was determined using a multivariable logistic regression model incorporating five potential predictive factors. Each significant variable was assigned a score based on a point scale, and the total probability of osteoporosis was estimated by summing these scores and mapping them onto a total point scale. As shown in Figure 3, this nomogram visually represents the predictive model, integrating clinical variables such as Tb.N value, waist circumference, BMI, sex, and age. Each variable is assigned a corresponding score based on its value, and the cumulative score is then matched to a probability scale at the bottom to estimate the likelihood of osteoporosis. For example, a 79-year-old male with a Tb.N value of 0.08, a waist circumference of 90 cm, and a BMI of 28.05 would receive approximately 1.1 point for Tb.N (Tb.N value = 0.08), 2.2 for waist circumference (90 cm), 6.9 for BMI (BMI = 28.5), 0 for sex (male), and 8 for age (79 year-old). The total score of 18.2 points corresponds to an estimated osteoporosis probability of approximately 45%.

## 4. Discussion

Osteoporosis is a systemic disease defined as a reduction in bone mass associated with an impaired bone architecture, increased bone fragility and increased fracture risk. The micro-architecture of trabecular bone is acknowledged as a key determinant of bone quality [8]. In healthy bone, trabeculae form a dense, interlacing lattice that efficiently distributes mechanical loads. Conversely, reductions in trabecular volume or thickness weaken this internal scaffold, diminish load-bearing capacity, and ultimately heighten fracture risk. This study shows that adding a single trabecular morphometric variable to a clinical model (age, sex, waist circumference and BMI) significantly improves discriminatory performance, with the area under the curve rising from 0.711 to 0.738 (*p* < 0.05). This result demonstrates that trabecular morphometry provides meaningful, incremental information for osteoporosis risk assessment.

Previous investigations have shown that osteoporosis is accompanied by changes in trabecular micro-architecture. Jin et al. analysed femoral specimens from an ovariectomised (OVX) mouse model of osteoporosis with micro-CT, comparing a normal control group (n = 10) with an OVX group (n = 46). BV/TV, Tb.Th, Tb.N, Tb.Sp and fractal dimension were calculated; all four morphometric indices differed significantly between groups (*p* < 0.05) [9]. In a clinical study of 108 men with lumbar osteopenia (mean age 52.1 years, T-score < −2.5), Legrand et al. found that, although BV/TV and Tb.Th did not have significant differences between 62 subjects with at least one vertebral compression fracture and 46 fracture-free controls, both Tb.Sp and Tb.N were significantly altered [10]. Majumdar et al. employed high-resolution magnetic resonance imaging at 1.5 T to acquire distal radius images in three female groups—premenopausal normal (n = 10), postmenopausal normal (n = 9) and postmenopausal fracture (n = 11). Compared with the postmenopausal-normal group, the postmenopausal fracture group showed significant differences in BV/TV, Tb.Sp and Tb.N. In addition, both Tb.Sp and Tb.N exhibited moderate correlations with trabecular BMD [11]. These studies highlight the diagnostic relevance of trabecular morphometry, yet they remain limited to animal models or small human cohorts.

More recently, LDCT—acquired for lung-cancer screening—has emerged as an opportunistic source of skeletal information. Pan et al. demonstrated that simple vertebral Hounsfield-unit (HU) thresholds at T11–L2 could identify low BMD and osteoporosis, and Marque et al. showed that serial LDCT HU values sensitively track two-year changes in vertebral trabecular density. These HU-based approaches demonstrated that LDCT images can be a tool for bone assessment [12,13]; however, they only quantify bone quantity and do not interrogate micro-architectural quality.

The present study analyzed a cohort of more than 400 participants, providing a larger-scale evaluation of trabecular morphometry in the context of osteoporosis. Our micro-architectural analysis showed no significant difference in BV/TV between osteoporosis and non-osteoporosis groups, whereas Tb.Th was lower in the osteoporotic group—an observation consistent with earlier reports [9]. In the present software, Tb.N is calculated according to the previously published formula [14,15]—as BV/TV divided by Tb.Th, a BV/TV that shows no significant change combined with markedly thinner trabeculae inevitably drives the ratio upward, producing an apparent increase in Tb.N. Rather than indicating a true gain in trabeculae, this elevation reflects the deterioration of the trabecular network—namely, thinning plates. For that reason, Tb.N was retained as a model variable: when interpreted in conjunction with BV/TV and Tb.Th, it serves as an integrated quantitative marker of micro-architectural compromise and adds discriminatory power to the multivariable analysis, with its Tb.N value yielding the highest AUC of 0.623. Fractal dimension captures the degree of irregularity and fragmentation in trabecular bone texture. Previous studies have shown that individuals with osteoporosis or osteopenia exhibit higher FD values than healthy controls, reflecting a more fragmented and complex trabecular architecture [16]. Consistent with these findings, the present study likewise observed a significantly higher FD in the osteoporotic cohort compared with the non-osteoporotic group.

We translated age, sex, BMI and LDCT-derived Tb.N into a nomogram that supplies an individualized probability of osteoporosis without extra imaging or radiation, thereby extending LDCT from HU-based bone-quantity screening to micro-architectural evaluation. Since 1 July 2022, Taiwan’s National Lung Cancer Early Detection Program has provided biennial LDCT to adults aged 45–74 years who are heavy smokers or have a lung cancer family history—an age range that overlaps the prime diagnostic window for osteoporosis [17]. In regions where osteoporosis remains under-detected, such as Taiwan [4,18], our LDCT nomogram delivers simultaneous, actionable information on lung cancer and skeletal health during the same visit.

A recent narrative review highlights that, with the aid of artificial intelligence, LDCT possesses considerable multifunctional potential [2]. By applying artificial intelligence algorithms to LDCT images, it is possible to not only predict the risk of lung cancer but also assess the risks of smoking-related comorbidities, such as cardiovascular disease and mortality. The present study provides evidence that the same LDCT scan can be used to extract Tb.N, which offers additional discriminatory value for assessing osteoporosis risk. With the aid of emerging artificial intelligence tools, LDCT holds forward-looking potential as an opportunistic screening modality that enables simultaneous evaluation of pulmonary, cardiovascular, and skeletal health in a single scan.

Our study has some limitations. First, this was a single-centre retrospective investigation, which may limit the generalizability of the findings to the broader population. Nevertheless, correlations between trabecular bone morphometric features and osteoporosis were identified in the relatively larger cohort. Second, several potentially relevant covariates—such as duration of comorbid conditions, medication history (e.g., glucocorticoids, hormone-replacement therapy)—were not systematically recorded. These factors can modulate bone density and micro-architecture and may have introduced residual confounding.

In addition, no subgroup analyses by sex or age groups were performed. These factors may influence the observed results and should be considered in future studies. Further studies with a comprehensive capture of relative variables are therefore warranted. Third, we acknowledge the computational nature of Tb.N and therefore avoid interpreting it as a direct biological indicator of the actual number of trabeculae. The observed increase in Tb.N should be interpreted with caution, as it may largely reflect reductions in trabecular thickness (Tb.Th) and related structural alterations rather than a true increase in trabecular elements. Although BV/TV was not statistically significant in our cohort, this may reflect the limited sample size and inherent variability in measurements. All measurement procedures followed standardized protocols to minimize potential bias. Fourth, the model did not undergo internal or external validation, which may limit the generalizability of the findings. Calibration and Decision Curve Analysis were not performed due to limited sample size, and future studies should assess clinical utility. In the limitations, we noted that multi-vertebral analyses in future studies may help validate the robustness and generalizability of the results. Fifth, the extremely large odds ratio for Tb.N (19,062.087) and its wide confidence interval indicate potential model instability, likely influenced by the variable scale and limited number of events.

In conclusion, our findings demonstrate that trabecular bone morphometry provides valuable information for assessing osteoporosis risk. When Tb.N extracted from routine LDCT scans is combined with age, sex, waist circumference and BMI, the ability to discriminate osteoporosis appears to improve modestly. The resulting nomogram offers an individualized probability of osteoporosis during the same LDCT scan that many adults already undergo for lung cancer screening, without requiring additional radiation or imaging time.

## Figures and Tables

**Table 1:** Patient clinical characteristics in the study cohort according to osteoporosis status (N = 429).

	Total, N = 429	Osteoporosis (−), N = 334	Osteoporosis (+), N = 95	*p*-Value
Gender				0.001
Female	134 (31.2%)	91 (27.2%)	43 (45.3%)	
Male	295 (68.8%)	243 (72.8%)	52 (54.7%)	
Age (years)	64.78 ± 8.271	63.83 ± 8.101	68.13 ± 8.030	<0.001
BMI (kg/m^2^)	25.187 ± 3.688	25.67 ± 3.567	23.51 ± 3.628	<0.001
Body fat percentage (%)	24.808 ± 7.156	25.01 ± 7.058	24.12 ± 7.482	0.287
Waist circumference (cm)	88.91 ± 9.652	89.99 ± 9.174	85.19 ± 10.359	<0.001
Blood urea nitrogen (mg/dL)	19.03 ± 9.264	18.88 ± 8.747	19.54 ± 10.941	0.543
Creatinine (mg/dL)	1.415 ± 0.662	1.42 ± 0.681	1.39 ± 0.595	0.683
eGFR (mL/min/1.73 m^2^)	51.649 ± 9.275	51.86 ± 8.919	50.90 ± 10.448	0.373
Smoking, n (%)				0.574
No or passive smoking	292 (68.1%)	223 (66.8%)	69 (72.6%)	
Quit smoking (at least one year)	70 (16.3%)	55 (16.5%)	15 (15.8%)	
Smoking	65 (15.2%)	54 (16.2%)	11 (11.6%)	
Alcohol drinking, n (%)				0.428
Alcohol drinking (−)	408 (95.3%)	316 (94.9%)	92 (96.8%)	
Alcohol drinking (+)	20 (4.7%)	17 (5.1%)	3 (4.4%)	
Exercise, n (%)				0.748
Exercise (−)	260 (63.1%)	202 (63.5%)	58 (61.7%)	
Exercise (+)	152 (36.9%)	116 (36.5%)	36 (38.3%)	
Cardiovascular disease, n (%)				0.197
No	387 (90.2%)	298 (89.2%)	89 (93.7%)	
Yes	42 (9.8%)	36 (10.8%)	6 (6.3%)	
Thyroid disease, n (%)				0.311
No	410 (95.6%)	321 (96.1%)	89 (93.7%)	
Yes	19 (4.4%)	13 (3.9%)	6 (6.3%)	
Cancer history, n (%)				0.572
No	401 (93.5%)	311 (93.1%)	90 (94.7%)	
Yes	28 (6.5%)	23 (6.9%)	5 (5.3%)	
Autoimmune disease, n (%)				0.291
No	421 (98.1%)	329 (98.5%)	92 (96.8%)	
Yes	8 (1.9%)	5 (1.5%)	3 (3.2%)	
Diabetes mellitus, n (%)				0.086
No	299 (69.7%)	226 (67.7%)	73 (76.8%)	
Yes	130 (30.3%)	108 (32.3%)	22 (23.2%)	

Missing data: Smoking (total: 2 cases, 0.5%; osteoporosis (−) group: 2 cases, 0.6%); Alcohol drinking (total: 1 case, 0.2%; osteoporosis (−) group: 1 case, 0.3%); Exercise (total: 17 cases, 4.0%; osteoporosis (−) group: 16 cases, 4.8%; osteoporosis (+) group: 1 cases, 1.1%). Abbreviations: BMI, body mass index; eGFR, estimated glomerular filtration rate.

**Table 2:** Trabecular bone morphometry analysis according to osteoporosis status.

Variable	Osteoporosis (−), N = 334	Osteoporosis (+), N = 95	*p*-Value
BV/TV	41.03 ± 4.287	41.41 ± 3.237	0.418
Tb.Th	1.53 ± 0.278	1.44 ± 0.194	0.004
Tb.Sp	1.89 ± 1.315	1.72 ± 0.246	0.222
Tb.N	0.27 ± 0.045	0.29 ± 0.036	0.001
D2D	1.65 ± 0.087	1.67 ± 0.068	0.015
D3D	1.83 ± 0.100	1.87 ± 0.085	0.003
QTS	19.17 ± 3.066	18.10 ± 2.756	0.002

Abbreviations: BV/TV, bone volume to total volume; Tb.Th, trabecular thickness; Tb.Sp, trabecular separation; Tb.N, trabecular number; D2D, 2D fractal dimension; D3D, 3D fractal dimension; QTS, quality of trabecular structure.

**Table 3:** Logistic regression models for prediction of osteoporosis.

Variable	Coefficient	OR	95% CI	*p*-Value
Model 1: Combined model (clinical and trabecular bone morphometry profiles)				
Age	0.068	1.07	1.037–1.104	<0.001
Gender	−0.561	0.571	0.325–1.002	0.051
BMI	−0.188	0.829	0.722–0.951	0.008
Waist circumference	0.019	1.019	0.967–1.075	0.478
Tb.N	9.855	19,062.087	22.233–16,343,266.88	0.004
Overall				
Model 1	5.702	299.455	63.133–1420.388	<0.001
Model 2: Clinical model				
Age	0.059	1.06	1.029–1.092	<0.001
Gender	−0.659	0.517	0.298–0.898	0.019
BMI	−0.184	0.832	0.727–0.952	0.007
Waist circumference	0.01	1.01	0.959–1.063	0.714
Overall				
Model 2	5.905	366.701	70.006–1920.821	<0.001

Abbreviations: BMI, body mass index; Tb.N, trabecular number; OR, odds ratio; CI, confidence interval.

**Table 4:** Evaluating osteoporosis predictive performance with models and trabecular bone morphometry.

Variable	AUC	95% CI	*p*-Value	
Tb.Th	0.362	0.299–0.425	<0.001	
Tb.N	0.612	0.549–0.675	0.001	
D2D	0.585	0.521–0.650	0.011	
D3D	0.609	0.546–0.671	0.001	
QTS	0.367	0.303–0.430	<0.001	
Model 1	0.738	0.680–0.796	<0.001	
Model 2	0.711	0.647–0.774	<0.001	
Comparison	Difference between areas	SE	95% CI	*p*-value
Model 1 vs. Model 2	0.0273	0.0119	0.00393–0.0506	0.022

Abbreviations: Tb.Th, trabecular thickness; Tb.N, trabecular number; D2D, 2D fractal dimension; D3D, 3D fractal dimension; QTS, quality of trabecular structure; AUC, area under the ROC curve; CI, confidence interval; SE, standard error.

## Data Availability

All data used for this study were obtained from anonymous medical records and are therefore not publicly accessible.
